# Bioallethrin enhances generation of ROS, damages DNA, impairs the redox system and causes mitochondrial dysfunction in human lymphocytes

**DOI:** 10.1038/s41598-021-87799-3

**Published:** 2021-04-15

**Authors:** Amin Arif, Ruhul Quds, Riaz Mahmood

**Affiliations:** Department of Biochemistry, Faculty of Life Sciences, Aligarh Muslim University, Aligarh, UP 202002 India

**Keywords:** Biochemistry, Environmental sciences

## Abstract

Bioallethrin is a synthetic pesticide that is widely used to control insect pests. The wide use of bioallethrin has resulted in inevitable human exposure. In this study we report the effect of different concentrations of bioallethrin (10 to 200 µM, 2 h at 37 °C) on human lymphocytes under in vitro conditions. Bioallethrin treatment resulted in loss of cell viability (> 30% at 200 µM bioallethrin). Oxidative stress markers like lipid peroxidation and protein oxidation were significantly increased accompanied by lower ratio of reduced to oxidized glutathione. Enhanced ROS generation was observed through fluorescence spectroscopy and microscopy. Bioallethrin-induced oxidative stress also compromised the antioxidant defence as it reduced antioxidant capacity of cells and inhibited major antioxidant enzymes. Biomolecular modifications and systemic toxicity by bioallethrin resulted in plasma membrane damage with mitochondrial depolarization. Comet assay showed nuclear DNA fragmentation and strand scission with significant increase in tail length and olive tail moment. Apoptosis and necrosis of cells was confirmed through acridine orange/ethidium bromide dual staining and visualization under fluorescence microscope. Thus, bioallethrin causes oxidative damage and compromises the antioxidant system leading to DNA damage, cellular and organelle toxicity, resulting in apoptosis and necrosis of human lymphocytes.

## Introduction

Vector borne diseases cause nearly 700,000 deaths annually of which more than 62% are attributed to mosquitoes on account of malaria and dengue, as reported by the World Health Organisation^1^. Pesticides like pyrethroids are widely used to control the spread of these diseases. Pyrethroids constitute a family of synthetic derivatives of pyrethrins that are classified as type I and II on the basis of absence or presence of cyano group, respectively.

Although pyrethroids were developed as relatively safe insecticides, yet theyare toxic to non-target species, like humans. However, their use is still prevalent worldwide and due to their wide applications, pyrethroids constitute about 25% of the total insecticide market. Their extensive use has resulted in significant exposure of a variety of populations to their harmful effects^2, 3^. Pyrethroids manifest an array of adverse effects^4^; these include hepato-, hemo-, neuro-, reproductive- toxicity and in many other systems^5–7^. In addition, DNA damage leading to carcinogenicity and mutagenicity has also been reported^8, 9^. Free radical generation, oxidative stress and alteration of antioxidant (AO) enzyme activities are a few of the known causesmediating the toxicity of pyrethroids^10, 11^. Allethrin is a pyrethroid that is neurotoxic to insects; it prolongs the opening of sodium channels which results in a hyperexcitable state leading to paralysis or death. Being the first synthesised pyrethroid, it has been used extensively for decades and researched well to explore its sequelae over non-target species. While its reproductive, developmental toxicity and neurotoxic potential are well established, biochemical and histological studies also showits adverse effects^12–14^. Allethrin causes mitochondrial mediated apoptosis in human corneal epithelial cells and is genotoxic in bacterial as well as mammalian systems^15, 16^. Allethrin biodegradation takes place by an oxidative pathway^17^.

Bioallethrin is a synthetic pyrethroid used as a pesticide against household pest insects such as mosquitoes, houseflies and cockroaches. It is a racemic mixture of two (R and S) out of eight stereoisomers of allethrin. Bioallethrin represents the most active stereoisomers of allethrin and is independently used in several insecticides. Despite its use in several household insecticides not much is known about its toxicity in human system. Research suggests bioallethrin causes behavioural and neurological damage and also functions as a potential endocrine disruptor^18–21^. In a study, S-bioallethrin exposure induced apoptosis in human lymphocytes while changing their gene expression profile^22^. In addition, bioallethrin was found positive in micronuclei assay in fish erythrocytes suggesting its genotoxic potential^23^.

Blood represents a major target of toxicants and xenobiotics since it is directly exposed to them and the fact that it is a connective and sentinel tissue. Pyrethrins are reported to cause anemia and synthetic pyrethroids have shown down regulation of several cytokines leading to impaired immune capacity^24, 25^.A study on influence of allethrin and prallethrin has shown altered plasma biochemical profiles in humans, and suggests a need of detailed toxicological studies on such pyrethroids^26^. Immunotoxicological potential of S-bioallethrin was shown by inhibition of human lymphocyte proliferation, in an in vitro study with basophils^27^.

Recently, we have reported the oxidative stress mediated effects of bioallethrinin human erythrocytes^28^. Erythrocytes are uniquely adapted for oxygen transport and have lost nucleus and organelles to enhance their oxygen carrying capacity. However, being non-nucleated cells, erythrocytes are not representative of the rest of the cells of the human body. Lymphocytes (unlike erythrocytes) are nucleated blood cells with other organelles which allow further studies on the effects of bioallethrin over human cellular system, especially on DNA and organelle damage. In this study the effect of bioallethrin has been examined using lymphocytes as the cellular system, We show that bioallethrin, in a dose-dependent manner, induces cell death, oxidizes proteins and thiols, lowers AO capacity, damages DNA and causes mitochondrial dysfunction in human lymphocytes.

## Results

### Cell viability

**Figure 1:**
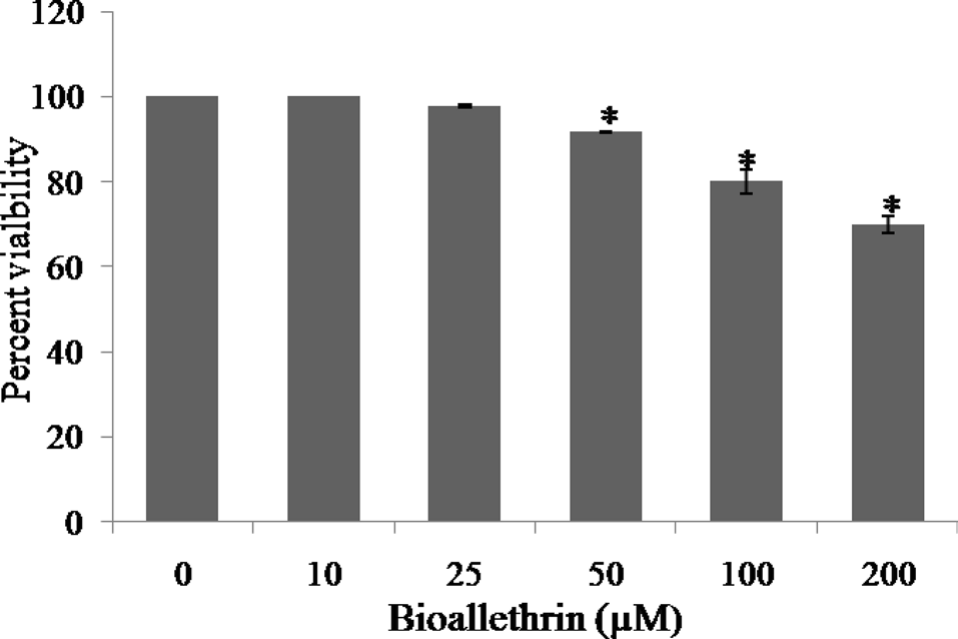
Effect of bioallethrin on viability of human lymphocytes. Cell viability was determined by the MTT assay as described in “Methods”. Absorbance of samples was read at 570 nm. Viability of control samples was taken as 100%. Lymphocytes were incubated with bioallethrin for 2 h at 37 °C. Results are ± standard deviation of six different samples. *Significantly different from control at p < 0.05. *MTT* 3-(4,5-dimethylthiazol-2-yl)-2,5-diphenyl tetrazolium bromide.

Cell viability of lymphocytes was determined by MTT assay from the absorbance of purple colored formazon. Cells exposed to the lowest concentration of bioallethrin (10 µM) remained viable for 2 h at 37 °C. However, a concentration dependent decrease in lymphocyte viability was evident at higher concentrations of bioallethrin. At 200 µM bioallethrin, the highest concentration used, almost 70% cells were viable (Fig. 1).

### Oxidative stress parameters

**Figure 2:**
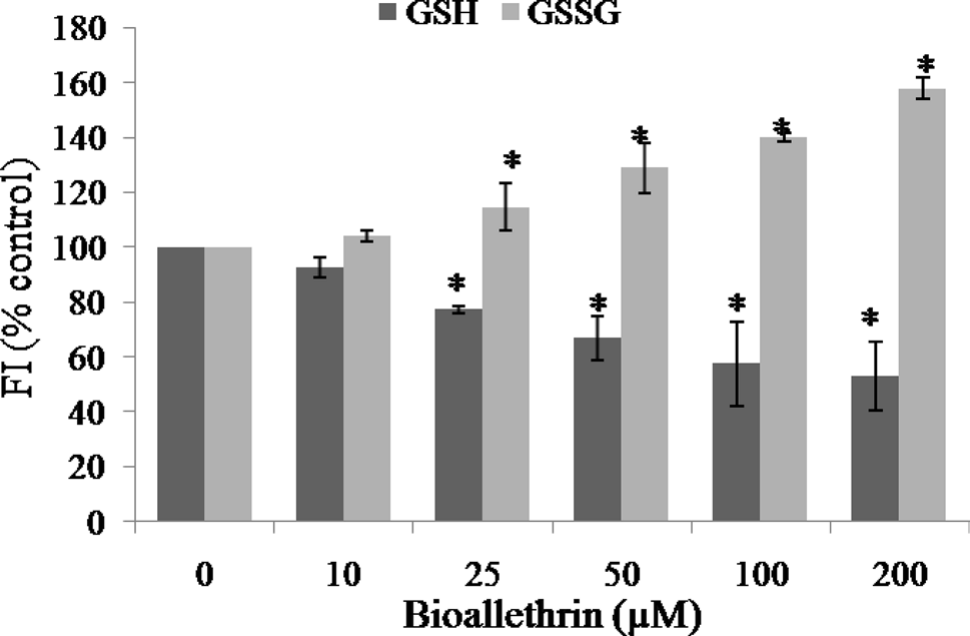
Effect of bioallethrin on reduced and oxidized glutathione levels. GSH and GSSG levels were determined fluorometrically in cell lysates and are shown as percent control, taking fluorescence of control sample as 100%. Lymphocytes were incubated with bioallethrin for 2 h at 37 °C. Results are ± standard deviation of six different samples. *Significantly different from control at P < 0.05. *GSH* reduced glutathione; *GSSG* oxidized glutathione; *FI* fluorescence intensity (arbitrary units).

A set of experiments based on oxidation of proteins, unsaturated lipids and thiols were used as indicators of oxidative stress condition in the cell. This included the determination of lipid peroxidation, protein oxidation, AOPP and glutathione (reduced/oxidised) in whole cell lysates. Lipid peroxidation and protein oxidation were doubled at the highest concentration of bioallethrin and AOPP was raised to 2.56 times, in comparison to control (Table 1). However, GSH was depleted to 53% while GSSG was raised to 1.6 fold in cell lysates of the lymphocytes treated with 200 µM bioallethrin, when compared to corresponding control values (Fig. 2).

**Table 1:** Effect of bioallethrin on oxidation of lipids and proteins.

[Bioallethrin]	MDA	PC	AOPP
Control	2.15 ± 0.09	55.57 ± 4.69	50.67 ± 3.36
10 μM	2.18 ± 0.14	56.53 ± 4.89	53.87 ± 2.69
25 μM	2.38 ± 0.02	62.30 ± 2.59*	59.31 ± 1.52
50 μM	2.56 ± 0.04*	75.11 ± 2.75*	80.84 ± 4.21*
100 μM	2.98 ± 0.12*	96.0 ± 3.94*	98.78 ± 2.42*
200 μM	3.97 ± 0.21*	114.87 ± 4.02*	129.82 ± 6.26*

All parameters were determined in cell lysates.

MDA concentration is in μmoles/mg protein while PC and AOPP are in nmoles/mg protein.

Lymphocytes were incubated with bioallethrin for 2 h at 37 °C.

Results are ± standard deviation of six different samples.

*MDA* malondialdehyde; *PC* protein carbonyls; *AOPP* advanced oxidation protein products.

*Significantly different from control at p < 0.05.

### Antioxidant capacity and antioxidant enzymes

**Figure 3:**
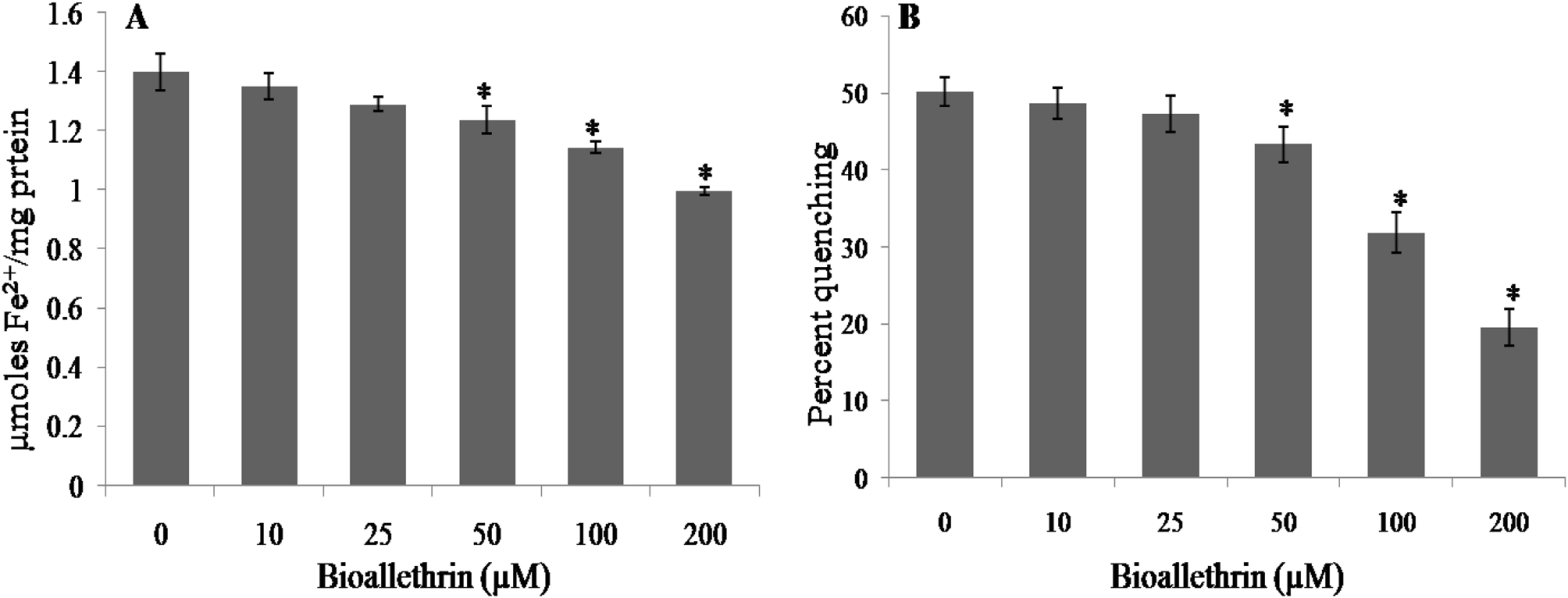
Effect of bioallethrin on antioxidant capacity of lymphocytes by (**A**) FRAP and (**B**) DPPH assays. FRAP values are in μmoles Fe^2+^/mg protein while DPPH results are given as percent quenching of DPP^**•**^ radical. Lymphocytes were incubated with bioallethrin for 2 h at 37 °C. Results are ± standard deviation of six different samples. *Significantly different from control at p < 0.05. *FRAP* ferric reducing ability/antioxidant power; Fe^2+^, ferrous; *DPPH* 2,2-diphenyl-1-picrylhydrazyl.

**Figure 4:**
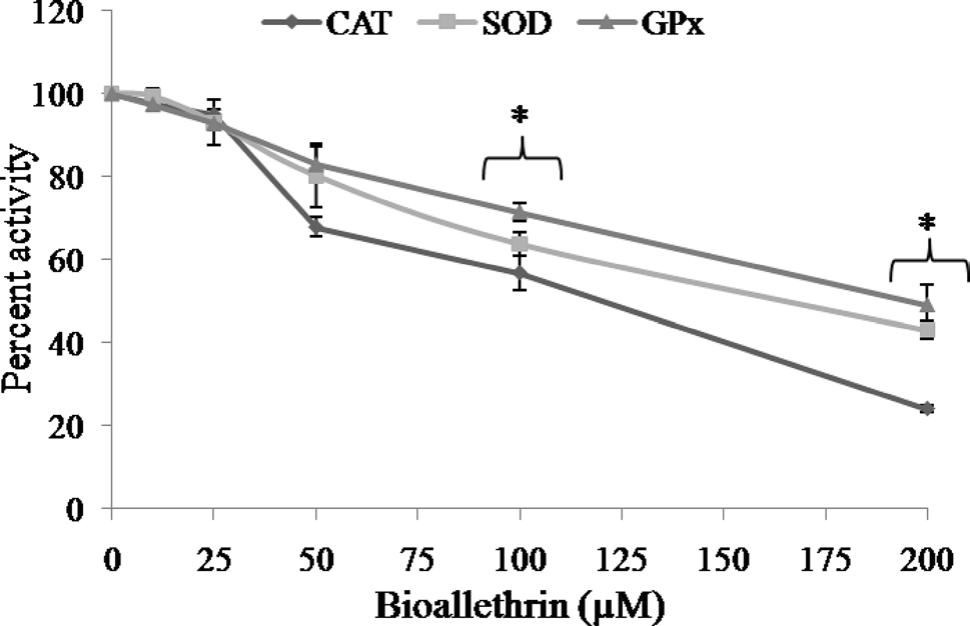
Effect of bioallethrin on the activities of antioxidant enzymes. All enzyme activities were determined in cell lysates. They are shown as percent activity with the activity of bioallethrin untreated (control) sample taken as 100 percent. Lymphocytes were incubated with bioallethrin for 2 h at 37 °C. Results are ± standard deviation of six different samples. *Significantly different from control at p < 0.05. *SOD* Cu,Zn superoxide dismutase; *CAT* catalase; *GP* glutathione peroxidase.

Antioxidant defence system of isolated human lymphocytes was monitoredin the form AO capacity and activities of AO enzymes. A decrease of about 30% in FRAP and a much greater 60% lowering in DPPH radical quenching was observed in 200 µM bioallethrin exposed samples when compared to control (Fig. 3). The results indicated a compromised AO defence system, hence major AO enzymes were assayed for further validation. In agreement, lower activities of enzymes (> 50%) were noticeable in a bioallethrin concentration dependent manner. At 200 µM bioallethrin, catalase was lowered dramatically and was less than 25% of control activity while SOD and GP were reduced to 43% and 49% of control, respectively (Fig. 4).

### ROS generation

**Figure 5:**
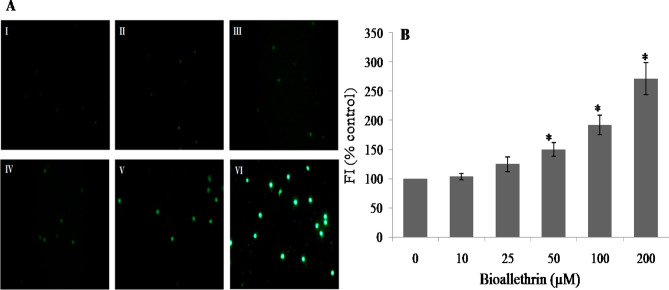
Effect of bioallethrin on ROS generation determined using DCFH-DA. (**A**) Fluorescence microscopic images showing lymphocytes at increasing bioallethrin concentration. I control sample; II–VI lymphocytes treated with 10, 25, 50, 100 and 200 µM bioallethrin, respectively (**B**) Fluorescence intensity of samples, shown in terms of percent control, taking fluorescence of control sample as 100%. Lymphocytes were incubated with bioallethrin for 2 h at 37 °C. Results are ± standard deviation of six different samples. *Significantly different from control at p < 0.05. *ROS* reactive oxygen species; *DCFH-DA* dichlorodihydrofluoresceindiacetate; *FI* fluorescence intensity (arbitrary units).

Positive and significant alterations in oxidative stress markers with compromised AO defence of cell strongly suggest enhanced generation of ROS in bioallethrin treated lymphocytes. This was studied by the widely used DCFH-DA method. DCFH-DA is a neutral and cell permeable molecule that freely diffuses across cell membranes. Inside the cell, esterases hydrolyze it to diacetate and DCFH; the latter is oxidized to highly fluorescent DCF by intracellular ROS. Higher fluorescence indicates more generation of ROS. An increase in the number of fluorescent cells was seen in bioallethrin treated lymphocytes when visualized under the fluorescence microscope (Fig. 5A). The fluorescence of cells was also quantified by a fluorometer. A similar result was seen, with bioallethrin exposed cells exhibiting higher fluorescence. In 200 µM bioallethrin treated cells, the fluorescence intensity was 2.71 times the control value (Fig. 5B). In both cases, the increase in fluorescence was in a bioallethrin concentration-dependent manner.

### Cell morphology

**Figure 6:**
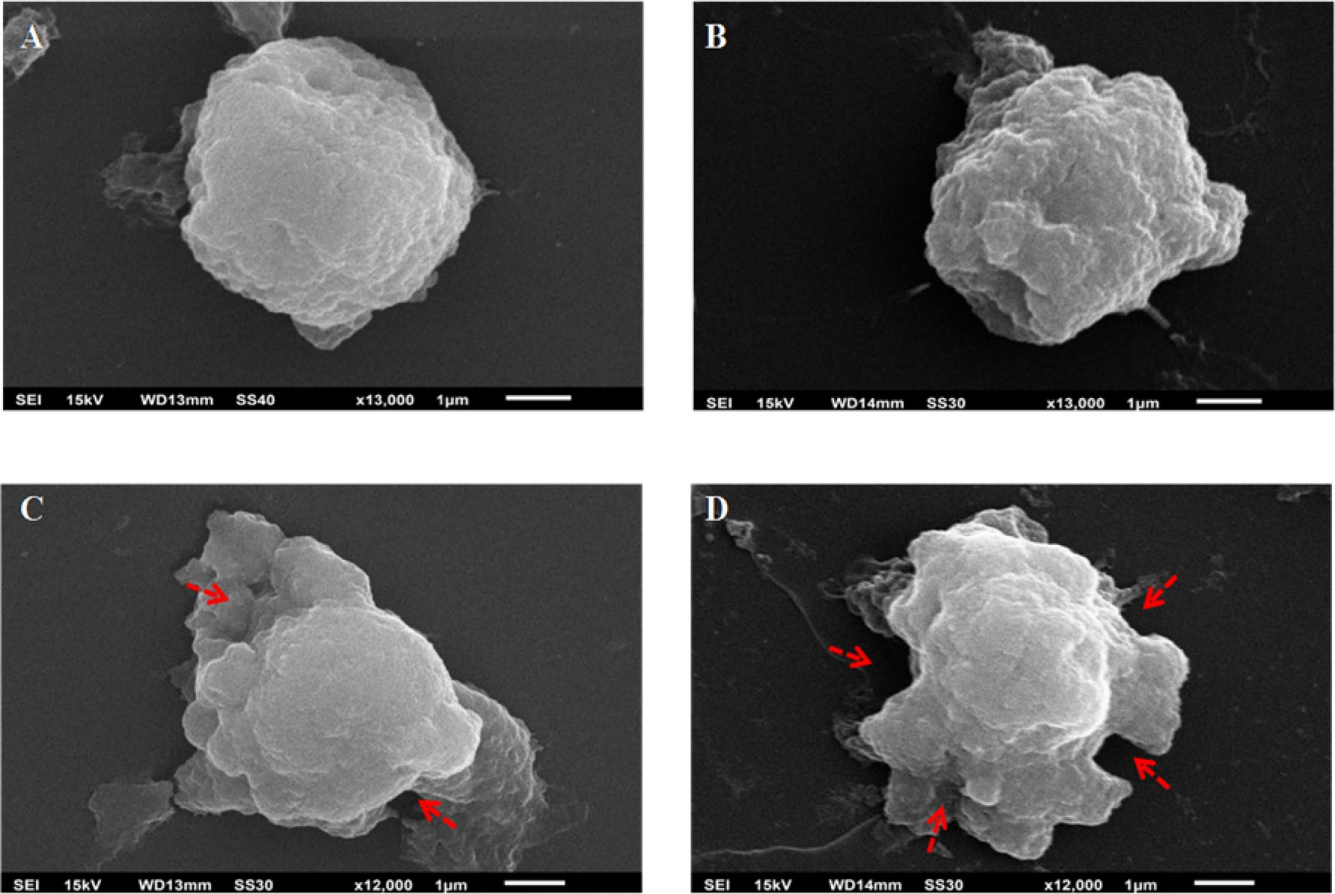
Scanning electron microscopy of lymphocytes. (**A**) Control (untreated) lymphocytes. Cells treated with (**B**) 50 µM (**C**) 100 µM and (**D**) 200 µM bioallethrin. Lymphocytes were incubated with bioallethrin for 2 h at 37 °C. Magnification is × 13,000 (**A**,**B**) and × 12,000 (**C**,**D**). Red arrows indicate the disrupted morphology of lymphocytes due to membrane damage.

Scanning electron microscopy was employed to detect morphological changes in isolated human lymphocytes exposed to bioallethrin. Evenly smooth and round lymphocytes were seen in bioallethrin untreated sample (control), which changed over different concentration in treated cells. Alterations in plasma membrane, uneven surface and distorted morphology was clearly visible in bioallethrin treated samples, especially at higher pesticide concentrations (100 and 200 µM) (Fig. 6).

### Mitochondrial membrane depolarisation

**Figure 7:**
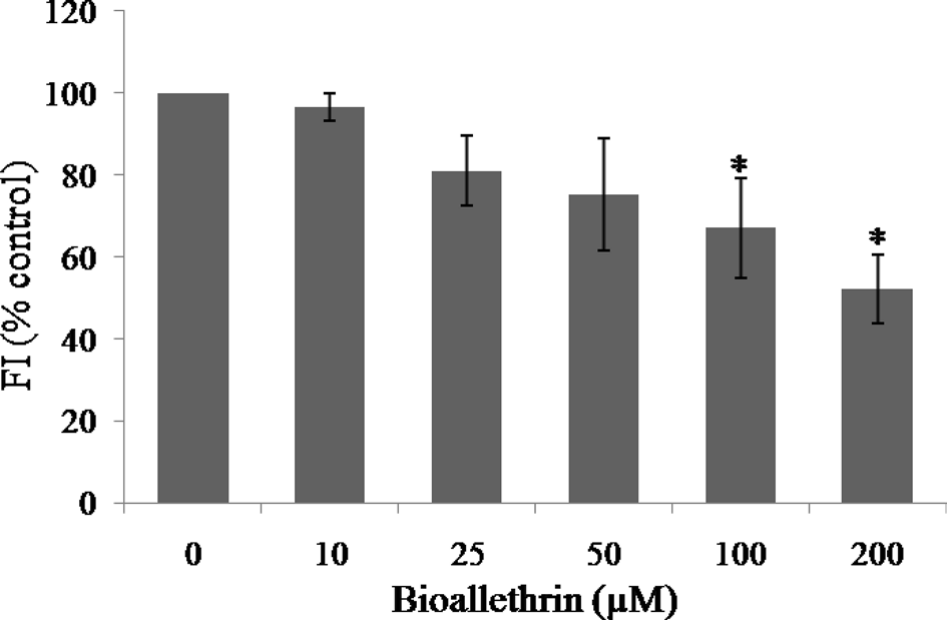
Effect of bioallethrin on mitochondrial membrane potential. Bioallethrin treated and control cells were mixed with Rhodamine 123 dye and fluorescence of samples recorded. Results are shown as percent control, taking fluorescence of control sample as 100%. Lymphocytes were incubated with bioallethrin for 2 h at 37 °C. Results are ± standard deviation of six different samples. *Significantly different from control at p < 0.05. *FI* fluorescence intensity (arbitrary units).

Rhodamine 123, a cationic dye, specifically labels the polarised inner mitrochondrial membrane which is a marker of respiring mitochondria. Loss of membrane potential results in loss of dye and fluorescence. The dye loaded lymphocytes were treated with increasing concentrations of bioallethrin to observe its effect on mitochondrial function. The membrane potential was found to be decreased in a concentration dependent manner and fluorescence in 200 µM bioallethrin treated lymphocytes was almost halved, relative to control (Fig. 7).

### DNA damage

**Figure 8:**
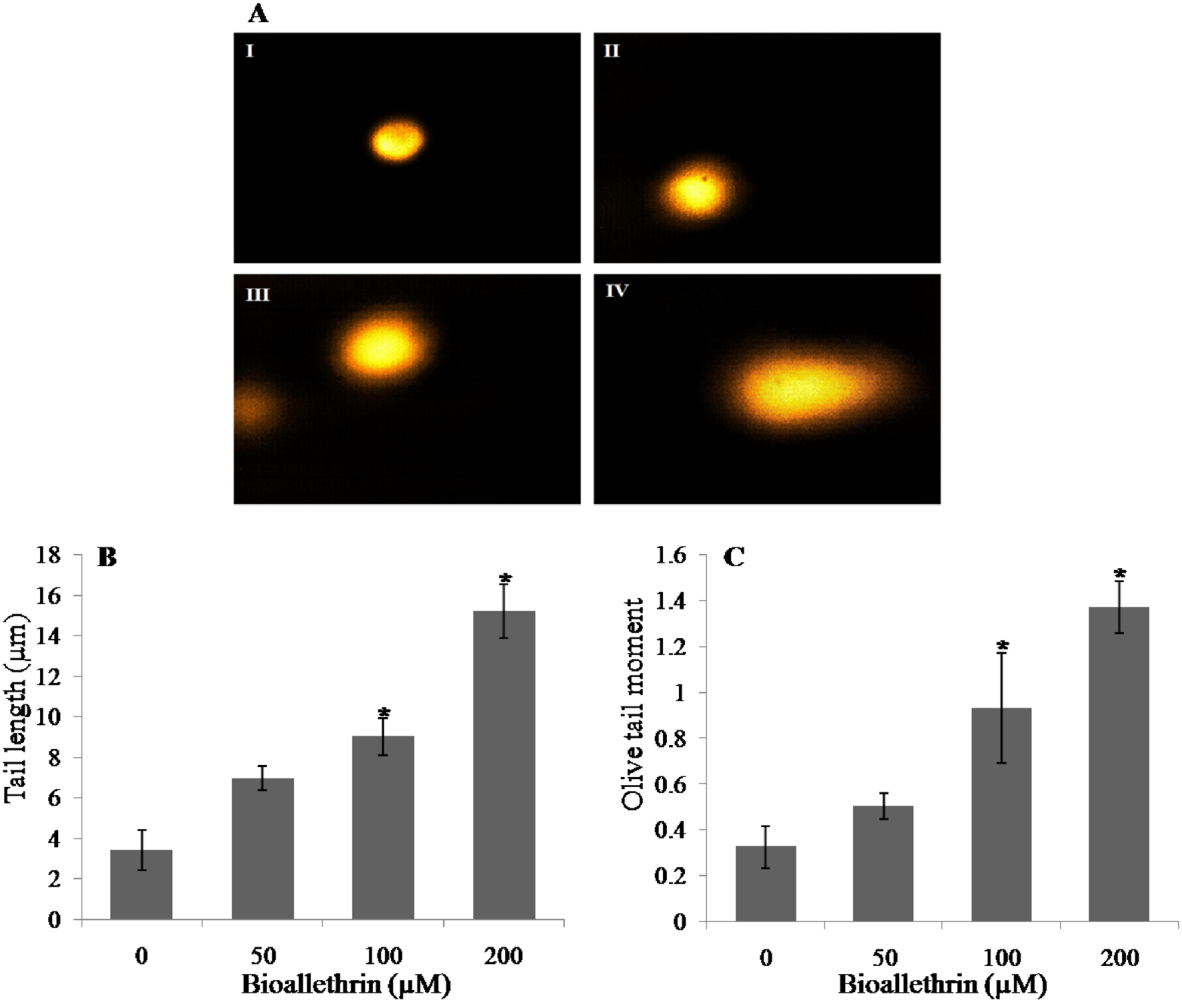
Effect of bioallethrin on nuclear DNA fragmentation examined by the comet assay. (**A**) The comet images are shown at 100 fold magnification (I) Control (untreated) lymphocytes. Cells treated with (II) 50 µM (III) 100 µM and (IV) 200 µM bioallethrin. (**B**) Comet tail length and (**C**) Olive tail moment. Lymphocytes were incubated with bioallethrin for 2 h at 37 °C. Data analysis was done by Komet 5.5, https://andor.oxinst.com/products/komet-software/komet-7. Results are ± standard deviation of six different samples. *Significantly different from control at p < 0.05.

Single cell gel electrophoresis (comet assay) was performed to decipher the genotoxicity of bioallethrin in terms of nuclear damage and DNA scission. Nuclear DNA strand scission, upon gel electrophoresis, generates a tail whose length is directly proportional to damage.Olive tail moment is the product of the tail length and the fraction of total DNA in the tail. In contrast to untreated cells, increasing concentrations of bioallethrin resulted in comets having longer tails showing concentration-dependent DNA damage (Fig. 8A). In addition, the comet tail length and olive tail moment were remarkably increased to more than four fold of control in 200 µM bioallethrin exposed lymphocytes (Fig. 8B,C).

### Nuclear morphology

**Figure 9:**
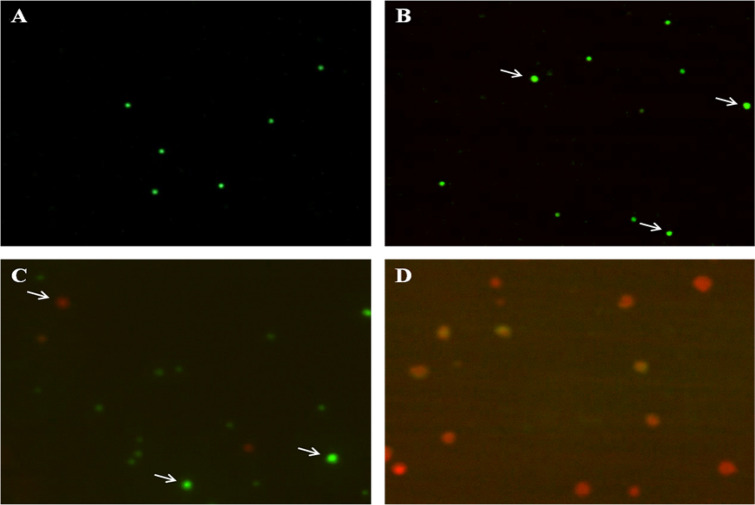
Effect of bioallethrin on nuclear morphology. Fluorescent images of cells double stained with acridine orange and ethidium bromide. (**A**) Control (untreated) and cells treated with (**B**) 50 µM (**C**) 100 µM and (**D**) 200 µM bioallethrin. Lymphocytes were incubated with bioallethrin for 2 h at 37 °C. Visual analysis was done by imageJ2, https://imagej.net/ImageJ2.

Mode of bioallethrin cell killing was investigated using acridine orange/ethidium bromide dual staining. Acridine orange, being a vital dye, stains all live as well as dead cells and ethidium bromide labels DNA only when the membrane integrity was compromised. After dual staining the circular green cells represent viable normal lymphocytes and bright yellowish green shows the early apoptotic ones. A partial dominance of red giving orange-yellow fluorescence marks late apoptotic cells, while full red dominating fluorescence signifies the necrotic cells. Untreated (control) sample has normal viable lymphocytes and exposure to 50 µM bioallethrin shows many of the cells entering early apoptotic phase (Fig. 9A,B). Further, the 100 µM bioallethrin exposure changes most of the normal cells to early apoptotic with a few late apoptotic as well as necrotic cells. The highest taken bioallethrin concentration (200 µM) brings necrosis to most of the exposed lymphocytes while inducing apoptosis into a few of them (Fig. 9C,D).

## Discussion

Human exposure to pesticides is a common phenomenon with consequences ranging from mild to severe health effects to death in extreme cases^29–31^. Serious concerns have been raised about health risks due to occupational exposure and from residues in food and drinking water. Annual pesticide use of over 2 million tonnes, with ever increasing demand worldwide, results in 3 million cases of pesticide poisoning and over 220,000 deaths annually^32–34^. The use of pyrethroids, like bioallethrin, in household products represents an even greater concern on account of their direct non-occupational exposure of the general population^35, 36^. To address the risk from these daily use chemicals it is important to understand their mechanism of toxicity so that methods can be devised to alleviate their toxic effects. To study the impact of a xenobiotic on human cells, lymphocytes are a feasible model in terms of their ease of isolation, processing and possessing fully functional organelles. They also yield results in quick time. Toxicity to isolated lymphocytes is also an indication to immunological damage by the compound under investigation, as reported by several researchers^37, 38^. Hence,the present study was designed to look at bioallethrin effect on oxidative damage, AO capacity, cell morphology, genotoxicity, organelle toxicity and apoptosis/necrosis has been performed using isolated human lymphocytes.

The MTT cell viability assay is commonly employed to quantify the damage done to selected mammalian cells^39^. Incubation of lymphocytes with bioallethrin led to the killing of significant amount of cells in limited time and 200 µM bioallethrin resulted in > 30% cell killing in 2 h. This clearly signifies the cytotoxic nature of this pyrethroid. A probable cause could have been the damage to membrane upon oxidation of unsaturated lipids and proteins. A known and major cause for such cell killing is oxidative stress which, above a threshold, is very damaging, even fatal, to any human/non-human cell^40, 41^. Several studies have reported that pyrethroids can induce oxidative stress condition leading to cell damage and death^42–44^. Well known markers of oxidative stress include monitoring lipid and protein oxidation and levels of glutathione (reduced and oxidised). In agreement with earlier studies, lipid peroxidation, protein carbonylation and AOPP were found to be increased significantly to suggest the oxidative mode of bioallethrin toxicity^45, 46^. The GSH content declined but it was accompanied by simultaneous increase in GSSG level. A decrease in GSH/GSSG ratio is a good indicator of oxidative stress^47^.

ROS are generated at low levels even under normal conditions, without xenobiotic stress, and all aerobic cells have a well developed check and balance system to counter them. This includes both enzymatic and non-enzymatic AO defence systems. Three major AO enzymes, which represent the prime and first line defence against oxidative stress, were assayed in control and bioallethrin treated lymphocytes^48^. Inhibition of catalase, SOD and GP confirms that exposure to bioallethrin impairs the enzymatic AO defence of lymphocytes causing oxidative stress and damage to the cells. Decreased activities of SOD and catalase will lead to accumulation of toxic levels of superoxide and H_2_O_2_, respectively^49^. Also, the depleted GSH (substrate to GP) levels on account of oxidative stress might be a cause of reduced GP activity. These three enzymes are also known to be inactivated by ROS^50, 51^. In view of these results it can be concluded that bioallethrin compromises both enzymatic (catalase, SOD, GP) and non-enzymatic (GSH) AO defence, leading to oxidation and damage of cell components.

The AO capacity of isolated human lymphocytes was monitored in control and bioallethrin exposed lymphocytes. Cellular AOs can donate an electron or H atom which can reduce metal ions or quench free radicals. This was measured by the widely used metal reducing (FRAP) and free radical quenching (DPPH) assays. Results of both assays clearly showed that bioallethrin reduces the AO capacity of treated cells in a concentration dependent manner. The inhibition of AO enzymes and low GSH level are likely responsible for this diminution in AO capacity which will make the cells more vulnerable to damage by ROS^52^. The result will be enhanced oxidation of biomolecules (proteins and lipids in this case) as seen here in bioallethrin treated cells.

Enhanced production of ROS is a major cause of oxidative stress which further damages plasma membrane together with vital organelles like mitochondria and nucleus leading to cell death^53, 54^. The oxidation of proteins, lipids and thiols together with inhibition of AO enzymes strongly suggested that bioallethrin is increasing intracellular ROS production in lymphocytes. The generation of ROS was confirmed and quantified by microscopic and fluorometric means, respectively. DCFH-DA assay clearly showed increase in ROS inside the cells when treated with different bioallethrin concentrations. Its impact on the plasma membrane was studied by scanning electron microscopy as a measure of cellular damage, as used earlier for lymphocytes and xenobiotics^55, 56^. Images of untreated (control) lymphocytes show the normal shape while bioallethrin treated cells clearly show increasing blebs and morphological deformities. Such morphological changes besides limiting the cell function, also make the cell vulnerable to organelle damage and even leads to death by necrosis^57, 58^.

Mitochondrial membrane potential (MMP) generated by proton pumps plays a vital role in energy storage and is harnessed to form ATP; fluctuations in MMP have deleterious effects on viability of cell^59^. Decreased MMP leads to the condensation of matrix and exposes cytochrome c to intermembranous space and the release of cytochrome c follows apoptotic cell death^60^. Due to its cationic nature, Rhodamine123 labels the mitochondria in intact living cells on the basis of mitochondrial potential. A significant decrease of MMP in bioallethrin treated cells was evident, which signifies the apoptosis derived death of isolated human lymphocytes. Oxidative stress is well studied to be related with mitochondrial dysfunction in human corneal endothelial cells as well as nuclear damage or genotoxicity in lymphocytes^61, 62^. Comet assay showed significant increase in tail length and olive tail moment which confirms nuclear damage and DNA scission in bioallethrin exposed lymphocytes. Genotoxicity caused by oxidative stress is well known cause of cell death; this may follow any of the two criteria of cell killing i.e. apoptosis or necrosis^63, 64^.

Higuchi (2003)^64^ described the possible involvement of oxidative stress and its markers with membrane damage, loss of mitochondrial membrane potential and nuclear damage; all leading to apoptosis or necrosis with slightly different mechanisms. Finally, contributions of both the possible cell death mechanisms were examined by employing acridine orange/ethidium bromide dual staining^65^. Results clearly suggest the role of apoptosis and necrosis together in bioallethrin induced cell death of isolated human lymphocytes via oxidative stress. At comparatively lower concentrations of bioallethrin (50, 100 µM) the cell killing was dominated by apoptosis while raised bioallethrin concentration (200 µM) leads to necrosis in most cells.

**Figure 10:**
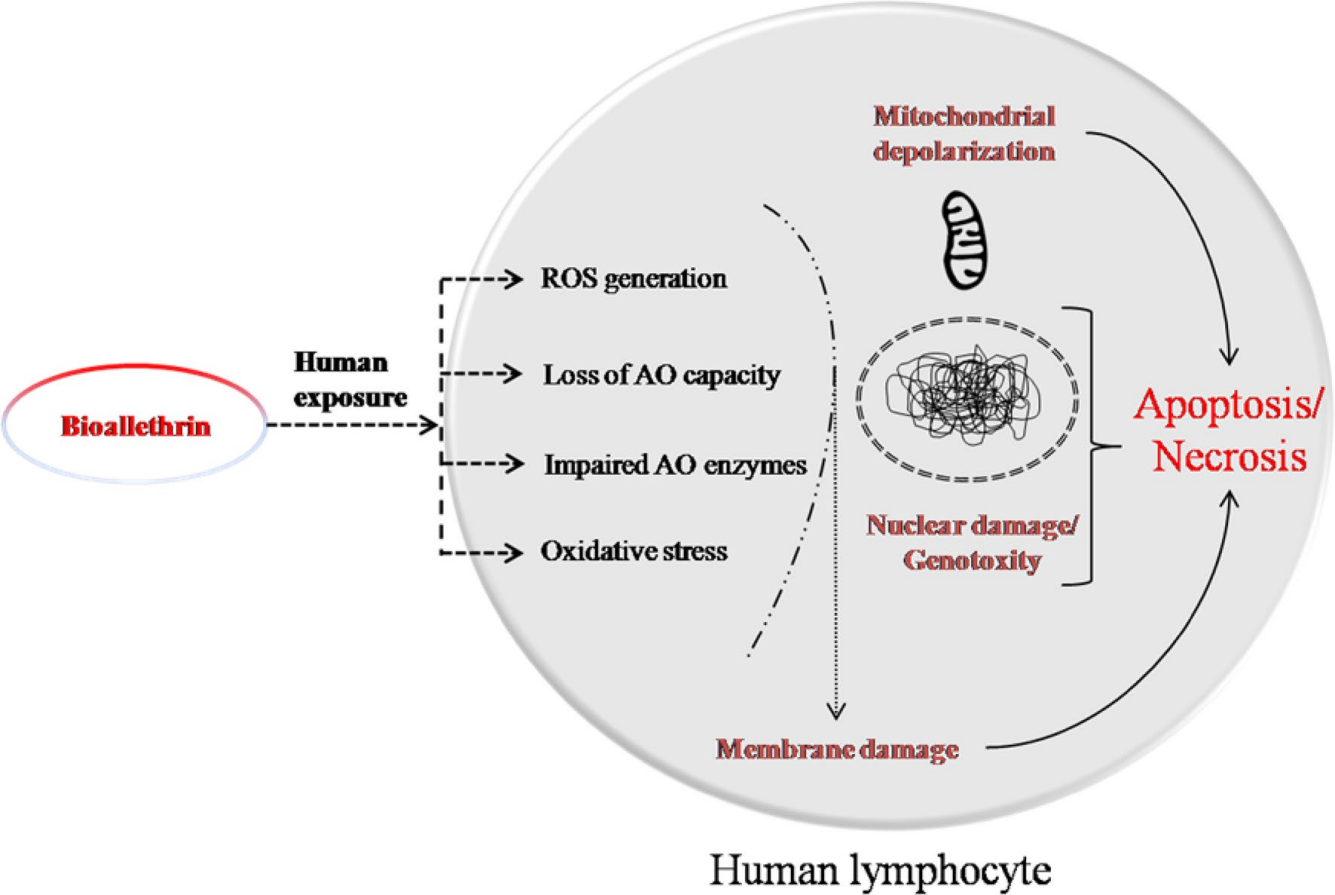
Schematic representation of the effects of bioallethrin on human lymphocytes. *ROS* reactive oxygen species; *AO* antioxidant.

A schematic representation of bioallethrin exposure to humans and its effects on human lymphocytes is shown in Fig. 10. Humans as a non target species get exposed to bioallethrin, when the household insecticide is used for the eradication of insects. This results in enhanced generation of ROS which oxidize proteins, lipids and thiols. It impairs the enzymatic and non-enzymatic AO defence systems and lowers AO capacity. This results in lymphocyte damage and modification of cell components. This bioallethrin induced ROS damage the membrane, alter cell morphology, depolarize mitochondrial membrane and also causenuclear DNA fragmentation. All this results in loss of human lymphocyte viability by apoptosis and necrosis in bioallethrin exposed cells.

In conclusion, bioallethrinexhibits cyto-, geno- and organelle- toxicity in human lymphocytes. It enhances the generation of ROS that impair the enzymatic as well as non-enzymatic AO defence of cell. The result is oxidative damage to cellular biomolecules like proteins, thiols and lipids. This oxidative damage extends to plasma membrane with vital organelles of the cell like nucleus and mitochondria. Limited use or very low concentrations of bioallethrin might be tolerated by the human defence mechanism(s), but higher concentrations of bioallethrin may result in cell death either by apoptosis or necrosis. Moreover, exposure to bioallethrin can be a cause of impaired immune system, as reported for other pyrethroids. The study provides a perspective to better understand the toxicity of bioallethrin and similar pyrethroids in humans. This will allow ways and methods to be devised that can alleviate the toxicity of this widely used pyrethroid.

## Methods

### Isolation of human lymphocytes, treatment with bioallethrin and preparation of cell lysates

This study was approved by the Institutional Ethics Committee of Aligarh Muslim University (Registration number 714/GO/Re/S/02/CPCSEA) that monitors research on human subjects. All methods were performed in accordance with the relevant guidelines. Healthy, 20–30 year old non-smoking volunteers, with no recent medication history, were used as donors after taking their informed and written consent. Blood was immediately mixed with equal volume of normal saline (0.9% sodium chloride) and layered over Histopaque 1077 in a 3:1 ratio. After centrifugation at 2400 rpm for 20 min the white buffy layer, just above Histopaque 1077, containing lymphocytes was removed. The cells were pelleted by centrifugation at 10,000 rpm, washed three times with normal saline and resuspended in saline to give a 10% (v/v) cell suspension. The lymphocyte suspensions were then incubated at 37 °C for 2 h with different concentrations (10, 25, 50, 100, 200 µM) of bioallethrin (Sigma-Aldrich, USA). A stock solution of 25 mM bioallethrin was prepared in DMSO and added to lymphocyte cell suspensions to get the desired bioallethrin concentration in the respective samples. Bioallethrin untreated cells served as control and were also kept at 37 °C for 2 h. Cell suspensions were centrifuged at 10,000 rpm for 10 min and pellets washed three times with normal saline. Finally, cell pellets were suspended in lysis buffer (0.2% Triton X-100, 100 mM NaCl, 1 mM EDTA and 20 mM Tris–HCl, pH 7.4), kept at 4 °C for 20 min and centrifuged again at 10,000 rpm for 10 min. Supernatants (cell lysates) were quickly frozen in aliquots at − 20 °C. Protein concentration in cell lysates was determined by the method of Lowry et al.^66^ using bovine serum albumin as standard.

### MTT assay

Cell viability was studied by the 3-(4,5-dimethylthiazol-2-yl)-2,5-diphenyl tetrazolium bromide (MTT) assay. A 5 mg/ml solution of MTT was prepared in phosphate buffered saline (PBS; 0.9% NaCl, 10 mM sodium phosphate buffer, pH 7.4) and 10 μl added to 1 ml of 10% suspension of bioallethrin treated and control cells. Samples were kept for 2 h at 37 °C, followed by centrifugation at 10,000 rpm for 5 min. After washing the pellets with PBS, 1 ml dimethyl sulfoxide was added to dissolve the blue formazan and absorbance recorded at 570 nm^67^.

### Oxidative stress markers

Several markers of oxidative stress such as malondialdehyde, carbonyl groups, advanced oxidation protein products (AOPP) and glutathione (reduced and oxidised, GSH and GSSG) were determined in cell lysates. The lipid peroxidation product malondialdehyde was determined from its reaction with thiobarbituric acid at 100 °C for 60 min and absorbance of the pink product formed was read at 532 nm^68^. Protein oxidation was followed from carbonyl content using 2,4-dinitrophenylhydrazine which reacts with carbonyl groups to give a hydrazone adduct that absorbs at 360 nm^69^. AOPP were determined by adding 0.2 M citric acid and 1.16 M potassium iodide to cell lysates and absorbance read at 340 nm; chloramine T was used as standard^70^. GSH and GSSG were determined fluorometrically using N-ethylmaleimide and o-phthalaldehyde, exactly as described by Hissin and Hilf (1976)^71^.

### Antioxidant capacity

Reduction of metal ion (Fe^3+^) and quenching of free radical (DPP^•^) were employed for the determination of AO capacity. Conversion of Fe^3+^ to Fe^2+^by AOs in sample and subsequent reaction of Fe^2+^ ions with 2,4,6-tris(2-pyridyl)-s-triazine form the basis of the ferric reducing antioxidant power (FRAP) assay^72^. Absorbance of the resulting blue complex was read at 593 nm. 2,2-Diphenyl-1-picrylhydrazyl (DPPH) in solution gives a stable purple coloured DPP^**•**^ free radical, which is converted to pale yellow DPPH by H donated by sample AOs. The decrease in absorbance of solution at 517 nm was recorded^73^.

### Antioxidant enzymes

All enzyme activities were determined spectrophotometrically in cell lysates. The specific activity of catalase was assayed from the decrease in absorbance at 240 nm upon decomposition of H_2_O_2_ into molecular oxygen and water^74^. Cu,Zn-superoxide dismutase (SOD) and glutathione peroxidase (GP) were assayed from inhibition of auto-oxidation of pyrogallol and oxidation of reduced nicotinamide adenine dinucleotide phosphate, respectively^75, 76^.

### ROS generation and mitochondrial membrane potential

Generation of reactive oxygen species (ROS) was monitored using dichlorodihydrofluoresceindiacetate (DCFH-DA) with the help of spectroscopy and microscopy^77^. Microscopic analysis and imaging were done by treating cell suspensions with bioallethrin as discussed in Sect. 2.1. Samples were centrifuged and pellets suspended in PBS to give 10% suspension and 10 μM DCFH-DA was added to 1 ml of each sample. About 80 µl suspension was placed over a slide and cells visualised under a fluorescence microscope (Olympus, Model BX43) using FITC filter.

The fluorescence of cells was quantified by recording it using fluorometer. Briefly, 10 μM DCFH-DA was added to 1 ml of 10% cell suspension. Cells were incubated for 1 h at 37 °C and then centrifuged at 10,000 rpm for 10 min and the supernatant, containing excess dye, was removed. A 10% suspension of the DCFH-DA loaded cells was prepared and treated with different concentrations of bioallethrin for 2 h at 37 °C. Appropriately diluted samples were excited at 485 nm and fluorescence emission recorded at 530 nm.

Mitochondrial membrane potential (MMP) was determined using fluorescent dye Rhodamine 123. Lymphocytes were processed as stated above for fluorometric determination except that 10 μM Rhodamine 123 was added instead of DCFH-DA. The fluorescence of samples was monitored for emission at 534 nm after excitation at 510 nm^78^.

### Scanning electron microscopy

Bioallethrin treated and control lymphocytes (prepared as described in “Cell viability”) were fixed with 2.5% glutaraldehyde for 2 h and then centrifuged at 10,000 rpm for 10 min. The lymphocyte pellets were washed and suspended in PBS to give 10% suspension. A drop of this cell suspension was placed on a glass slide and dried at 37 °C. Samples were then dehydrated with increasing concentrations of ethanol in distilled water reaching 100% for the last wash^79^. Dehydrated lymphocytes were gold coated and observed under scanning electron microscope (SEM).

### Comet assay

Genotoxic potential of bioallethrin was evaluated by the comet assay of Singh et al.^80^ with some modifications. Control and bioallethrin treated lymphocytes were incubated at 37 °C for 2 h, centrifuged and washed as described in “Cell viability”. The washed cell pellets were suspended in normal saline and mixed with equal volume of 0.5% low melting point agarose. Frosted glass slides were coated with 1% agarose and 80 µl cell suspension was evenly layered on each slide. Cells were lysed and DNA allowed to unwind by dipping the slides in alkaline solution (1 mM Na_2_-EDTA, 300 mMNaOH, pH 13.0) for 30 min at 4 °C. This was followed by electrophoresis for 20 min at 25 V and 300 mA. After electrophoresis, the slides were neutralised with 0.4 M Tris, pH 7.5, buffer and DNA stained with 20 µl of 20 µg/ml ethidiumbromide. The slides were gently washed with water to remove excess ethidium bromide, kept in a dark box and visualised under a fluorescence microscope (CX41, Olympus, Japan). Nuclear DNA damage/DNA strand scission was assessed by scoring the images. The images were scored and analyzed for comet tail length and olive tail moment using Komet 5.5 software (USA).

### Acridine orange and ethidium bromide staining

The lymphocytes were isolated and treated with different bioallethrin concentrations as described in “Cell viability”. The control and bioallethrin treated samples were washed and suspended in normal saline containing 100 µg/ml each of acridine orange and ethidium bromide^81, 82^. Then 80 µl of each dual stained lymphocyte sample was placed over a glass slide and covered with a glass slip. Cells were visualised and images for both fluorescent dyes were taken at exactly same locations by changing filters and later merged with ImageJ software. Merged images were analysed, sorted and labelled in reference to nuclear damage and mode of cell death.

### Statistical analysis

Reproducibility of the data was confirmed by taking blood samples from six different individuals. Error values in graphs and table are the standard deviation which depicts the experimental dispersion of the set. ANOVA and post-hoc test by Microsoft Excel were used to see the significance of the experimental results; a probability level of P ≤ 0.05 was considered significant and is denoted by asterisk (*) symbol.
